# Accuracy Enhancement in Refractive Index Sensing via Full-Spectrum Machine Learning Modeling

**DOI:** 10.3390/bios15090582

**Published:** 2025-09-05

**Authors:** Majid Aalizadeh, Chinmay Raut, Morteza Azmoudeh Afshar, Ali Tabartehfarahani, Xudong Fan

**Affiliations:** 1 Department of Biomedical Engineering, University of Michigan, Ann Arbor, MI 48109, USA; maalizad@umich.edu (M.A.); afarahan@umich.edu (A.T.); 2 Department of Electrical Engineering and Computer Science, University of Michigan, Ann Arbor, MI 48109, USA; 3 Center for Wireless Integrated MicroSensing and Systems (WIMS2), University of Michigan, Ann Arbor, MI 48109, USA; 4 Max Harry Weil Institute for Critical Care Research and Innovation, University of Michigan, Ann Arbor, MI 48109, USA; 5 Department of Computational Medicine and Bioinformatics, University of Michigan, Ann Arbor, MI 48109, USA; craut@umich.edu; 6 Informatics Institute, Istanbul Technical University, Istanbul 34485, Turkey; morteza.azmudeh@gmail.com

**Keywords:** machine learning, meta-grating, index sensor, linear regression, mean squared error

## Abstract

We present a full-spectrum machine learning framework for refractive index sensing using simulated absorption spectra from meta-grating structures composed of titanium or silicon nanorods under TE and TM polarizations. Linear regression was applied to 80 principal components extracted from each spectrum, and model performance was assessed using five-fold cross-validation, simulating real-world biosensing scenarios where unknown patient samples are predicted based on standard calibration data. Titanium-based structures, dominated by broadband intensity changes, yielded the lowest mean squared errors and the highest accuracy improvements—up to an 8128-fold reduction compared to the best single-feature model. In contrast, silicon-based structures, governed by narrow resonances, showed more modest gains due to spectral nonlinearity that limits the effectiveness of global linear models. We also show that even the best single-wavelength predictor is identified through data-driven analysis, not visual selection, highlighting the value of automated feature preselection. These findings demonstrate that spectral shape plays a key role in modeling performance and that full-spectrum linear approaches are especially effective for intensity-modulated index sensors.

## 1. Introduction

Resonant nanophotonic structures are widely used in optical biosensing, where changes in the surrounding refractive index produce measurable shifts in the optical spectrum [1,2,3,4,5,6,7,8,9,10,11,12,13,14,15,16,17,18]. These spectral changes are typically monitored using one of two sensing modalities: (1) tracking the wavelength shift in narrow resonance peaks [19,20,21,22,23,24,25,26,27,28], or (2) monitoring the intensity change at fixed wavelengths [29,30,31,32,33]. Most existing methods apply simplified one-dimensional fitting routines that rely on a visually selected spectral feature from one of these modalities. While practical, these approaches often overlook rich spectral information distributed across the full wavelength range, which can be potentially used for enhanced accuracy in sensing.

Machine learning has been increasingly used across scientific domains, including biosensing, to uncover patterns in complex data and enhance predictive performance [34,35,36,37,38,39,40,41]. Recent studies have shown that machine learning can significantly enhance biosensor performance, such as in wearable photonic wristbands for cardiorespiratory monitoring and biometric identification [42], and in bioreceptor-free biosensors where ML improves detection accuracy and specificity [39]. In our prior work [43], we introduced a machine learning approach that used multiple pre-selected resonance peak shifts as inputs for ridge regression. That method significantly reduced mean squared error in refractive index prediction, outperforming conventional single-feature techniques by orders of magnitude. However, it still required manual feature selection and was limited to spectra with well-defined peaks.

In parallel, the advancement of optical sensing hardware has been accelerated by the development of metamaterials. These are artificially structured materials engineered to manipulate electromagnetic waves beyond the capabilities of naturally occurring media [44]. Metasurfaces represent the two-dimensional class of metamaterials and offer powerful control over phase, amplitude, and polarization [45,46,47,48,49]. Among them, meta-gratings are periodic metasurfaces with a wide range of applications and are highly suited for biosensing applications due to their strong spectral sensitivity and tunability [50,51,52].

In this work, we explore whether full-spectrum machine learning, without manual feature selection or peak tracking, can improve prediction accuracy across both sensing modalities. We simulate a meta-grating structure consisting of triangular nanorods on a gold reflector, using either titanium (Ti) or silicon (Si) as the rod material. Ti produces broadband intensity modulation representative of fixed-wavelength sensing, while Si supports narrow Mie-type resonances with wavelength shifts. To account for the anisotropic response of the structure, we simulate both TE and TM polarizations. Cross-validation is used to evaluate generalization across unknown index values, mimicking practical biosensing scenarios.

To evaluate generalization, we apply five-fold cross-validation by holding out different refractive index values during training. This mimics experimental biosensing scenarios, where standard samples form a calibration curve and unknown analytes are predicted. Traditional methods rely on one-dimensional fitting at a visually selected wavelength, but our results show that even the best single predictor is identified through machine learning rather than visual inspection. Full-spectrum modeling with principal component analysis (PCA) and linear regression consistently improves accuracy, with up to a 6000-fold mean squared error (MSE) reduction in Ti-based structures due to their smooth, intensity-driven spectra. These findings underscore the critical role of spectral shape and highlight the advantage of full-spectrum approaches, particularly for meta-gratings governed by broadband intensity modulation.

## 2. Proposed Structure

**Figure 1:**
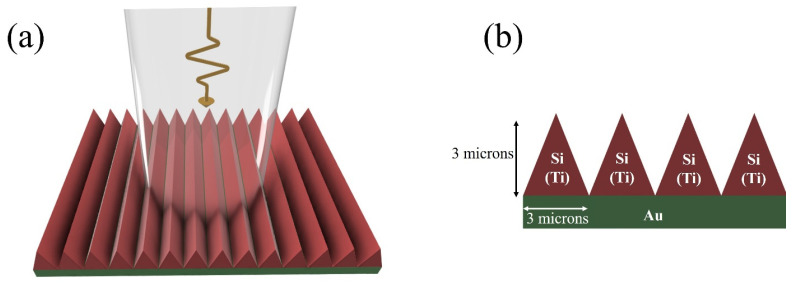
(**a**) Schematic and (**b**) cross-sectional side view of the meta-grating refractive index sensor consisting of triangular periodic nanorods on a gold back reflector. Both Si and Ti nanorods were simulated. The geometry serves as a unified platform to compare intensity-modulated (Ti) and resonance-shift (Si) spectral responses under TE and TM polarizations.

Figure 1 illustrates the meta-grating structure used as the bulk index sensing platform in this study. The geometry consists of a one-dimensional periodic array of triangular cross-sectioned nanorods positioned on an optically thick gold (Au) reflector. The simulations have revealed that other metals can be used as the back reflector as well, given that they are optically thick to block the transmission. Each nanorod has a base (period) and height of 3 μm, forming an isosceles triangular cross-section. The material of the nanorods is set to either Ti or Si, allowing for the exploration of different resonance behaviors. Ti supports broadband intensity-modulated spectra, while Si enables narrowband Mie-type resonances with distinct peak shifts. This design serves as a unified geometry for comparing different spectral modalities within the same structural platform.

In practice, achieving flat sidewalls in triangular nanorods requires precise fabrication. This can be accomplished either through etching, which often involves optimization of etch parameters, or through nanoimprint lithography, where the fabrication of the initial mold requires high precision. Such fabrication tolerances may influence the resulting optical response compared to the idealized simulations.

## 3. Simulation Results

**Figure 2:**
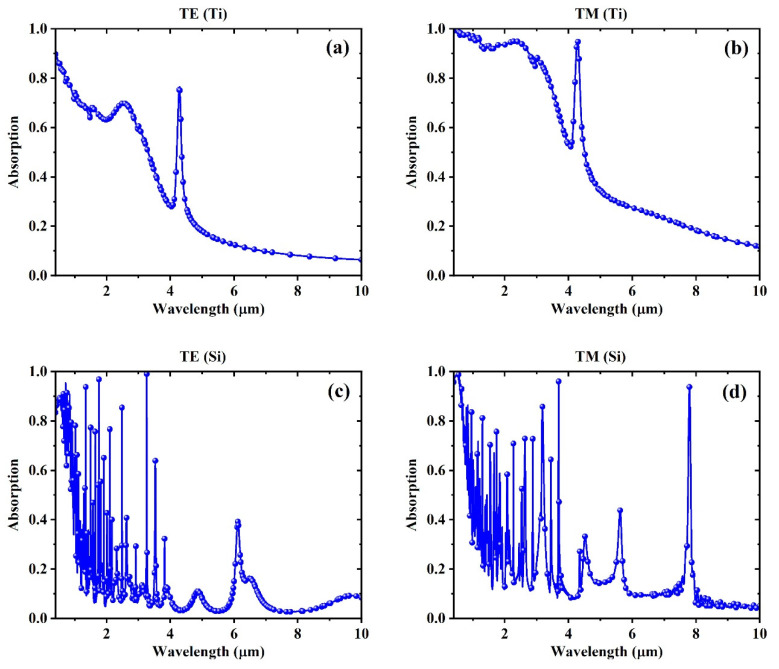
Absorption spectra of the structure at the environment refractive index of 1, using (**a**) Ti with TM (**b**) Ti with TE, (**c**) Si with TM, and (**d**) Si with TE polarization.

Figure 2 presents the absorption spectra of the meta-grating for different material and polarization combinations, with the refractive index of the environment fixed at 1. Figure 2a,b correspond to the Ti-based meta-grating under TM and TE polarizations, respectively. Both configurations display smooth, broadband absorption profiles with low spectral feature density. The spectra lack sharp resonances and instead show gradual intensity variations across the wavelength range. This behavior is characteristic of lossy metallic nanostructures and suggests that the optical response is dominated by broadband absorption rather than discrete resonance effects.

Figure 2c,d show the Si-based meta-grating under TM and TE polarizations, respectively. In these cases, the spectra exhibit a series of narrow, well-resolved resonances, indicative of Mie-type modes supported by high-index dielectric materials [43]. The spectral profiles show greater feature density and sharper variations compared to the Ti cases, and the position and spacing of the resonances differ slightly between TE and TM modes due to the anisotropic response of the structure. These differences in spectral shape across materials and polarizations set the foundation for evaluating how spectral modality influences modeling strategies in subsequent analyses.

**Figure 3:**
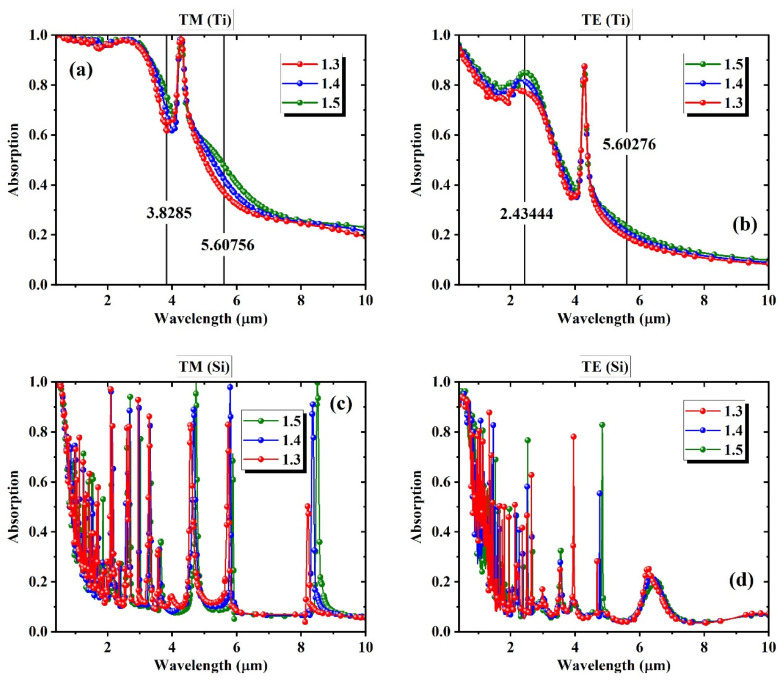
Absorption spectra of the structure at the environment with varying refractive indices of 1.3, 1.4, and 1.5 using (**a**) Ti with TM, (**b**) Ti with TE, (**c**) Si with TM, and (**d**) Si with TE polarization.

Figure 3 shows how the absorption spectra change with variations in the refractive index of the surrounding environment, ranging from 1.3 to 1.5. The meta-grating is simulated under TM and TE polarizations for both Ti and Si nanorods. Figure 3a,b correspond to the Ti-based structures, where the overall spectral shape remains consistent across different refractive indices. The dominant change is in the intensity of absorption at specific regions, rather than shifts in spectral position. This is more evident by the only resonance almost not shifting at all by varying the index. A few wavelengths that appear to exhibit relatively strong monotonic intensity changes are marked on the plots. These points represent wavelengths with the most prominent index-dependent intensity changes and are typically chosen in intensity-modulation–based biosensing approaches.

Figure 3c,d show the Si-based spectra under TM and TE polarizations. Unlike the Ti cases, the spectra here contain dense, sharp resonances that shift in wavelength as the refractive index varies. These features reflect Mie-type behavior typical of high-index dielectric nanostructures. The field analysis representing the physical phenomena underlying such resonances is thoroughly discussed in our prior work [43]. Because the peak shifts are narrow and localized, the intensity at most fixed wavelengths remains relatively constant unless a resonance aligns with that location. This creates a more complex spectral behavior that is less compatible with simple linear modeling. By contrast, the Ti spectra display smoother and more predictable global changes in intensity, which are better suited for linear regression. These observations establish the foundation for comparing full-spectrum machine learning performance across different structural and spectral regimes.

**Figure 4:**
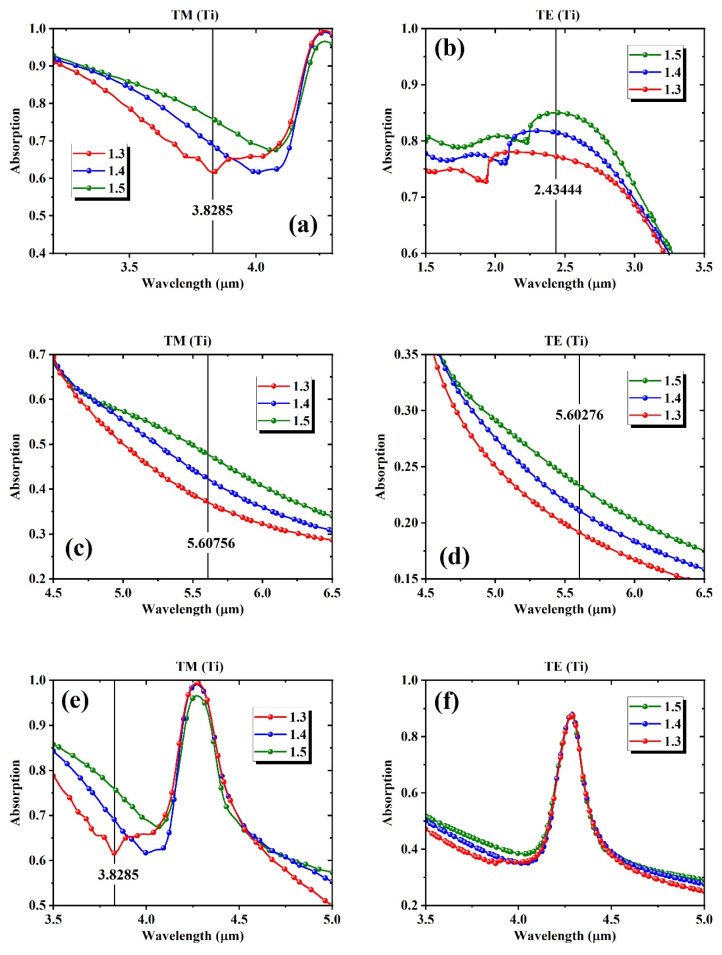
Localized absorption intensity variation in Ti-based meta-grating structures under TM (**left**) and TE (**right**) polarizations for refractive index values of 1.3, 1.4, and 1.5. (**a**–**d**) Spectra at selected fixed wavelengths showing clear intensity variation with refractive index. (**e**,**f**) Resonant regions illustrating nearly no spectral peak shift despite index variation, consistent with intensity-based modulation in Ti-based structures.

Figure 4 provides magnified views of the selected spectral regions from the Ti-based meta-grating, focusing on wavelengths with high variations in intensity across refractive indices. Figure 4a–d show the spectral response at four different wavelengths, chosen based on visually identifiable intensity changes across refractive indices. These wavelengths were selected because they appear to vary in a monotonic and relatively consistent manner, which aligns with the assumptions of linear modeling. Figure 4a,c show TM polarization results, while Figure 4b,d correspond to TE polarization. Each of these points is used to assess how well a single-wavelength intensity value can serve as a predictor for refractive index using conventional regression.

Figure 4e,f present the same Ti-based spectra, zoomed in around the single spectral peak in TM and TE polarizations, respectively. These plots demonstrate that, unlike the Si-based structures, the single peak position does not shift noticeably with changes in refractive index. The spectral shape remains fixed, and the only observable variation is in the amplitude of absorption. This highlights a key distinction in the sensing behavior of the Ti structure. While the intensity-based features allow for straightforward linear modeling, there is no resonant wavelength tracking involved. These observations confirm that intensity modulation is the dominant mechanism for index detection in Ti-based configurations.

**Figure 5:**
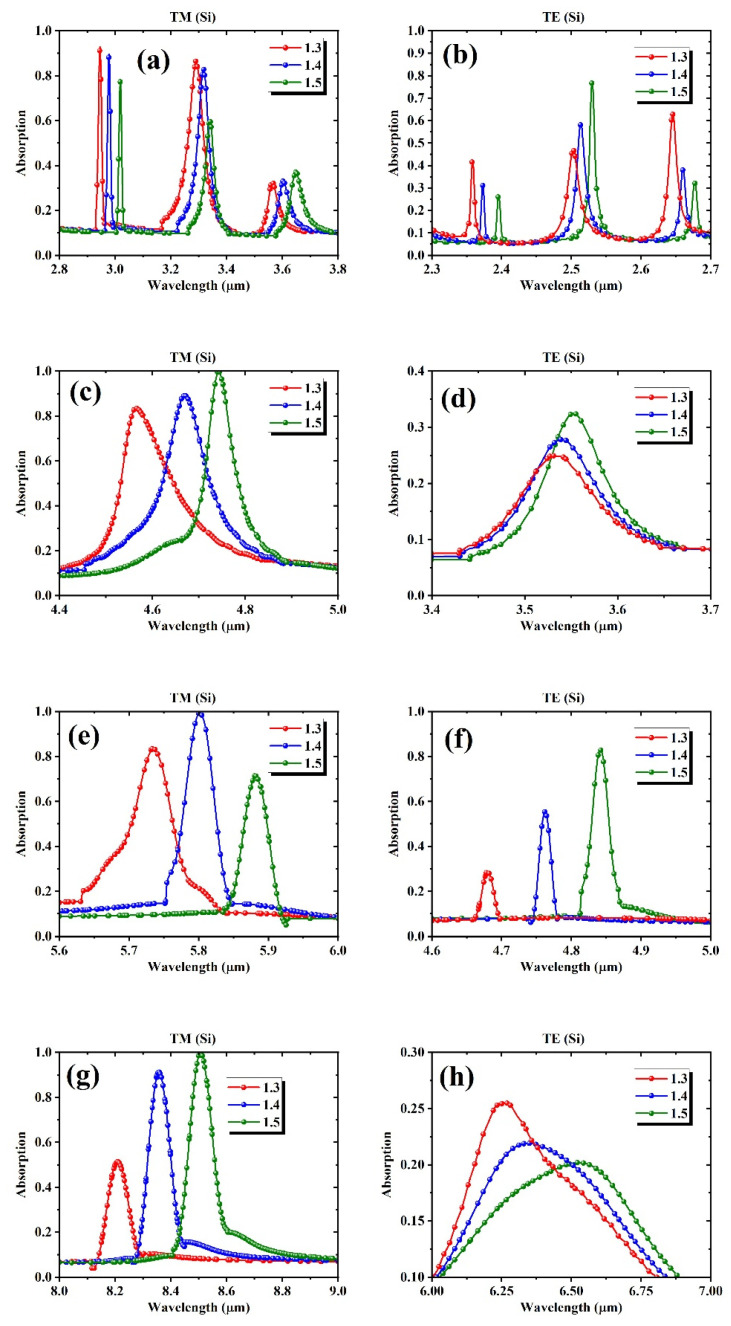
Localized resonance shifts in Si-based meta-grating structures under TM (left) and TE (right) polarizations for refractive index values of 1.3, 1.4, and 1.5 (**a**–**h**) Zoomed-in spectra at different wavelength ranges showing index-dependent peak shifts for TM (**a**,**c**,**e**,**g**) and TE (**b**,**d**,**f**,**h**) polarizations.

Figure 5 presents a series of zoomed-in absorption spectra for the Si-based meta-grating under TM and TE polarizations, illustrating sharp resonances that shift significantly as the bulk refractive index changes from 1.3 to 1.5. Each subpanel highlights one or more narrow Mie-type resonances and their red-shift across the index increase. The TM mode spectra are shown in the left panel, or Figure 5a,c,e,g, while TE mode results are given in the right panel, or Figure 5b,d,f,h. The selected peaks exhibit high spectral sensitivity and sharpness, with shifts that span tens of nanometers or more for a 0.1 variation in the bulk index. This results in nm/RIU in the order of tens. These shifts often vary in slope and symmetry depending on the peak’s location and the surrounding spectral context.

**Figure 6:**
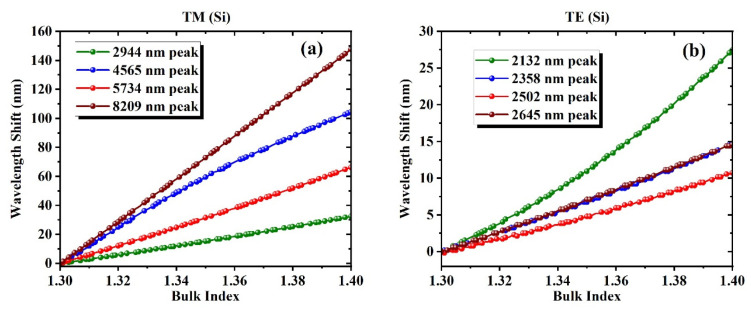
Peak location shift vs. bulk index in Si-based meta-grating under (**a**) TM, and (**b**) TE polarizarions. Linear behavior is evident while the 8209 nm peak in (**a**) demonstrates the most linear behavior and the least MSE. Our prior work showed that combining the peaks results in accuracy improvement up to 3 orders of magnitude.

These peak shifts, which occur consistently across a range of wavelengths, form the foundation of traditional biosensing approaches that rely on tracking the movement of a single resonance. Figure 6 further quantifies this behavior by plotting the wavelength shifts in selected resonances in the Si-based meta-grating as a function of bulk refractive index, revealing strong linearity for individual peaks. The TM polarization results are displayed in Figure 6a, and TE results are shown in Figure 6b. For both polarizations, the tracked peaks exhibit consistent redshifts as the refractive index increases from 1.30 to 1.40, confirming their suitability for refractive index sensing based on spectral displacement. The shift trends appear approximately linear over the simulated range for most peaks, although the magnitude of sensitivity varies substantially across resonance locations. Notably, the 8209 nm peak in the TM configuration shows both the largest and most linear shift, which, as will be discussed later, results in the lowest mean squared error among all single-predictor models in our regression analysis. This observation reinforces the strong link between linearity of spectral displacement and predictive performance in linear modeling frameworks.

When the peak tracing behavior is similar to the one shown in Figure 6, the 1-dimensional linear fitting for a selected peak is the traditional approach for the estimation of the unknown bulk index. In our previous work, we demonstrated that this linear response enables effective modeling through linear or ridge regression when multiple peak shifts are used as inputs, resulting in substantial accuracy improvements over single-feature methods. However, while these isolated peaks behave linearly with respect to index, the full spectrum, comprising many sharp, nonlinear resonances, does not. This fundamental mismatch between spectral shape and linear model assumptions limits the benefit of applying full-spectrum linear regression in the Si-based cases. As we will observe in later sections, unlike our prior study where linear models could leverage the additive effects of multiple linearly shifting features, the current full-spectrum approach yields modest enhancements in silicon structures due to the global nonlinearity embedded in their resonant response.

Table 1 summarizes the one-dimensional linear regression performance for selected peaks shown in Figure 6, based on their index-dependent shifts. For each peak, the MSE mean and MSE standard deviation for the hold-out sets across five-fold cross-validation are reported. The peaks are chosen from both TM and TE polarization modes of the Si-based structure. Notably, the 8209 nm peak under TM polarization demonstrates the lowest MSE (0.0294 ± 0.0068), indicating a highly linear relationship with refractive index (see Figure 6a). In contrast, some TE-polarized peaks, particularly at shorter wavelengths, exhibit higher MSE values, highlighting their limited utility for accurate linear modeling. These results reinforce the importance of feature selection when using single-variable models and provide a benchmark for evaluating data-driven multivariate approaches.

## 4. Machine Learning Based Full Spectrum Modeling for Precision Enhancement

**Figure 7:**
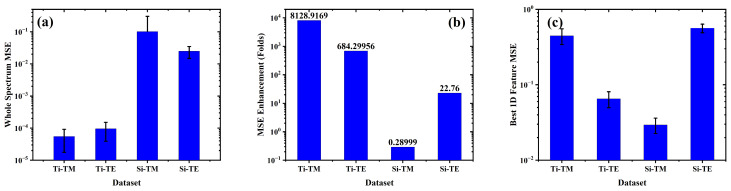
Modeling performance across datasets: (**a**) Full-spectrum MSE using up to 80 PCA components, (**b**) fold improvement over best single-feature fit, and (**c**) MSE of best single-feature model (peak shifts for Si). Error bars indicate standard deviation from five-fold cross-validation.

Figure 7 summarizes the modeling performance across the four datasets by comparing (a) the mean squared error (MSE) of the full-spectrum linear regression model using up to 80 principal components, (b) the corresponding fold enhancement in MSE relative to the best-performing single-feature linear fit, and (c) the MSE values for those best single predictors. The best single predictors were identified via exhaustive search; further details are described in the Supplementary Materials. These comparisons are made in light of the distinct spectral profiles observed in earlier figures. We also decided to use all 80 principal components during our model comparisons to ensure that we would be testing the effectiveness of incorporating all data provided by the spectrum and to ensure that between model variability is representative of differences in dataset and not model parameters such as how many and which principal components were included (Supplementary Table S3). When we tested the model performance using reduced numbers of principal components, we observed a rapidly decreasing benefit of incorporating additional principal components (Supplementary Figure S1 and Supplementary Materials).

In Figure 7a, Ti-based structures (Ti–TM and Ti–TE) exhibit extremely low MSEs of 5.49 × 10^−5^ and 9.53 × 10^−5^, respectively, when using full-spectrum modeling with up to 80 PCA components. These values are orders of magnitude lower than those of the Si-based structures: 0.101 for Si–TM and 0.0247 for Si–TE. This performance discrepancy directly reflects the underlying spectral behavior that Ti spectra exhibit smooth, broadband intensity variations with minimal resonant structure, allowing PCA-based linear regression to effectively capture the index-dependent variation. In contrast, Si spectra are dominated by sharp Mie-type resonances that make the intensity shift nonlinearly with index, making them less compatible with global linear modeling.

Figure 7b illustrates the fold enhancement in MSE obtained by transitioning from the best single-feature linear model to full-spectrum PCA-based regression. The Ti–TM dataset shows an 8128-fold reduction in error, followed by Ti–TE with a 684.3-fold improvement. For the Si–TE datasets, the enhancement is more modest (22.76) and for Si–TM dataset, the MSE has gotten worse compared to best 1D peak tracing case. This reflects the fact that much of the predictive information in resonance-dominated spectra is already concentrated in a small number of well-aligned peak features.

Figure 7c shows the baseline MSEs of the best individual peak predictors: 0.446 for Ti–TM, 0.0652 for Ti–TE, 0.0294 for Si–TM, and 0.5629 for Si–TE (see Table 1). Notably, while Si–TM yields the lowest single-feature error among the four, its full-spectrum model improves only marginally. This suggests that, despite spectral richness, the nonlinear behavior of its intensity variation limits the effectiveness of linear PCA-based modeling. In contrast, Ti-based datasets benefit substantially from this approach due to their linear and distributed spectral response.

To validate the use of linear regression, a comparison was performed using multiple machine learning algorithms, including Lasso, support vector regression, random forest, and gradient boosting, on the Ti–TE dataset. Linear regression achieved the lowest MSE and was therefore selected for all four datasets to ensure consistency and interpretability. Although the comparison was limited to a representative dataset, the spectral behavior and resulting trends across configurations confirm the appropriateness of the chosen model.

**Figure 8:**
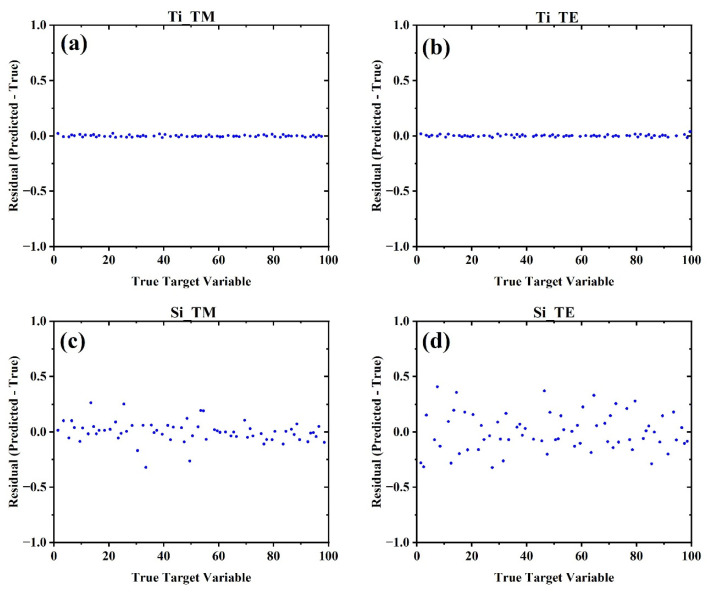
Residual plots from five-fold cross-validated full-spectrum linear regression. Titanium-based datasets (**a**,**b**) show minimal error, while silicon-based cases (**c**,**d**) exhibit larger, more scattered residuals.

Figure 8 presents the residual plots of the full-spectrum principal component regression (PCR) model across all four datasets, plotted as the difference between the predicted and true target variables against the true target values (index samples from 1.3 to 1.4 with 0.01 steps mapped onto 1 to 101 with 1 seps). These visualizations offer a direct interpretation of prediction error distributions and provide further insight into the compatibility between spectral behavior and linear modeling.

Figure 8a,b, corresponding to Ti–TM and Ti–TE datasets, respectively, reveal extremely tight clustering of residuals around zero, with minimal dispersion and no noticeable bias or systematic trend across the index range. This strongly supports the earlier findings of exceptionally low MSE in these configurations and reinforces that broadband, smooth intensity modulations in Ti-based spectra align well with linear PCA modeling. The residuals appear nearly uniform and homoscedastic, indicating high model fidelity throughout the entire sensing range.

In contrast, Figure 8c,d, associated with Si–TM and Si–TE datasets, show broader distributions of residuals, with Si–TE in particular displaying a visibly higher variance. This pattern is consistent with the elevated MSEs observed in Figure 7a and is attributed to the nonlinear intensity-shift behavior inherent to Mie-resonant metasurfaces. The increased variability reflects the challenge of approximating such nonlinearity using a limited number of principal components and a linear regression framework.

Together, these residual plots validate the earlier conclusion: the efficacy of linear modeling is heavily influenced by the underlying spectral transformation mechanism. For Ti metasurfaces with gradual, linear intensity shifts, PCR with up to 80 components offers precise, unbiased predictions. For Si metasurfaces, we also tested a series of nonlinear models (Supplementary Table S4). We observed that for the all the nonlinear models, the default hyperparameters resulted in larger mean MSE for the hold-out set in 5-fold cross-validation; however, with careful hyperparameter tuning it was possible for the Support Vector Regressor (SVR) with radial basis function to achieve similar performance to the linear regression for the Si metasurfaces.

It is also noteworthy that while laboratory systems can readily control TE or TM polarization, integrated or portable devices may benefit from designs that are less polarization sensitive. In future work, combining measurements from multiple polarizations, beyond the two principal TE and TM cases, could provide higher data density and improved precision depending on the application.

## 5. Conclusions

This study investigated the performance of full-spectrum machine learning models, specifically principal component analysis followed by linear regression, in predicting refractive index changes from simulated absorption spectra of metasurface-based biosensors. By comparing four datasets representing different material (Si, Ti) and polarization (TE, TM) configurations, we demonstrated that the predictive accuracy of linear models is strongly influenced by the nature of the underlying spectral changes. Ti-based meta-gratings, characterized by broadband intensity modulation with minimal spectral peak shifts, yielded exceptionally low mean squared errors (MSEs) and tightly clustered residuals. In contrast, Si-based structures with sharp nonlinear Mie resonances showed limited enhancement in performance when modeled using the same linear framework.

The results emphasize the importance of spectral shape in determining the compatibility of linear models with optical sensing data. While PCA-based regression is highly effective for intensity-modulated spectra, its performance is comparatively limited for spectra dominated by resonance shifts. Additionally, the large fold enhancements, exceeding 6000 times for the Ti-based structure and TM polarization, underscore the value of utilizing the entire spectral information rather than relying solely on isolated peak positions. These findings support the use of data-driven feature selection, even in traditional one-dimensional fitting approaches, and suggest that future work incorporating nonlinear modeling could further improve the interpretability and accuracy for complex resonance-based systems. While our results are based on simulated spectra, experimental implementations may face additional challenges such as random noise, temperature drift, and spectrometer resolution. These effects could influence the generalizability of full-spectrum models. Future studies may therefore benefit from incorporating noise reduction techniques, robust regression models, or improved hardware optimization to maintain prediction accuracy in real-world conditions.

## Figures and Tables

**Table 1:** Linear regression 1-dimensional fitting metrics for the peak shifts in Figure 6.

TM_Si			TE_Si		
Peak Location (nm) ^1^	MSE		Peak Location (nm)	MSE	
	Mean	std		Mean	std
**2944**	0.79198	0.16526	2132	10.035007	1.84389
**4565**	5.89753	1.07706	2358	1.822854	0.59077
**5734**	0.75589	0.10943	2502	2.907157	0.63826
**8209**	0.02944	0.00683	2645	0.562897	0.07325

Note ^1^: The peak locations (in nm) mentioned in the table refer to their locations at the bulk index of 1.3.

## Data Availability

The raw data supporting the conclusions of this article will be made available by the authors on request.

## Supplementary Materials

The following supporting information can be downloaded at: https://www.mdpi.com/article/10.3390/bios15090582/s1, Figure S1: Contribution of additional principal components to the hold-out MSE across 5-fold cross validation; Table S1: Training and test split statistics across 5-fold cross-validation for all datasets, including *p*-values for independence tests between splits. Table S2: Exhaustive single-wavelength predictor search results (10,000 features) with cross-validated hold-out MSE values for each dataset. Table S3: Contribution of additional principal components on hold-out MSE across the four datasets, showing optimal PC counts for each case. Table S4: Non-linear model performance (default and optimized hyperparameters) compared to linear regression across all datasets.
