# Supplementary material for: Accuracy Enhancement in Refractive Index Sensing via Full-Spectrum Machine Learning Modeling
Source: Biosensors (Basel). 2025 Sep 5;15(9):582. doi: 10.3390/bios15090582 (PMC12467005; doi:10.3390/bios15090582)
Supplement: Supplementary file 1 [file biosensors-15-00582-s001.zip › biosensors-3768411-supplementary.pdf]

# Supplementary Information for Accuracy Enhancement in Refractive Index Sensing via Full-Spectrum Machine Learning Modeling

Majid Aalizadeh[1,2,3,4], Chinmay Raut[5], Morteza Azmoudeh Afshar[6], Ali Tabartehfarahani[1,3,4], and Xudong Fan[1,3,4,*]

[1]Department of Biomedical Engineering,
University of Michigan, Ann Arbor, MI 48109, USA

[2]Department of Electrical Engineering and Computer Science,
University of Michigan, Ann Arbor, MI 48109, USA

[3]Center for Wireless Integrated MicroSensing and Systems (WIMS[2]),
University of Michigan, Ann Arbor, MI 48109, USA

[4]Max Harry Weil Institute for Critical Care Research and Innovation,
University of Michigan, Ann Arbor, MI 48109, USA

[5]Department of Computational Medicine and Bioinformatics,
University of Michigan, Ann Arbor, MI 48109, USA

[6]Informatics Institute
Istanbul Technical University, 34485 Istanbul, Turkey

*: Corresponding author: xsfan@umich.edu

# Cross-validation

Five-fold cross-validation was implemented through Python scikit-learn. For each of the 101 absorption spectra each was assigned a cross-validation fold from 1 to 5 inclusive forming 5 subsets of the data. For each data subset the absorption spectra and values were split into 5 bins containing 21, 20, 20, 20, and 20 values, respectively. Across 5 iterations, one partition was sequentially selected to be the hold-out set and models were trained from scratch using the remaining 4 partitions as training data. Since new models were fit on each fold of the data, we do not expect any data leakage with our protocol. Supplemental Table 1 demonstrates the training and test split mean and standard deviation for the target outcome and the 10,000 different predictors across each of the 5 folds for the 4 different material and polarization combination datasets. P-values represent a t-test of independence across the training partition and the test partition assuming unequal variances. Although data was not stratified, by studying the p-values for the target outcomes and predictors across the training and test splits for each of the 5 cross-validation folds, we do not find any evidence of imbalance or divergence in training and test split after accounting for the multiple testing burden.

# Identifying optimal single-wavelength predictors

To identify the best single-wavelength absorption spectra predictor for each of the datasets we performed an exhaustive search. For each of the 10,000 possible wavelength predictors we fitted a linear regression model and evaluated the performance of the single-variable model on the hold-out set following the cross-validation protocol described previously. We ranked each predictor based on the descending order of mean hold-out MSE. Results for all single-variable testing can be found in Supplementary Table 3. It is noteworthy that for Si datasets, 1D peak tracing is compared to ML approaches, the values of which are shown in Table 1 of the main manuscript.

# Selecting the number of principal components

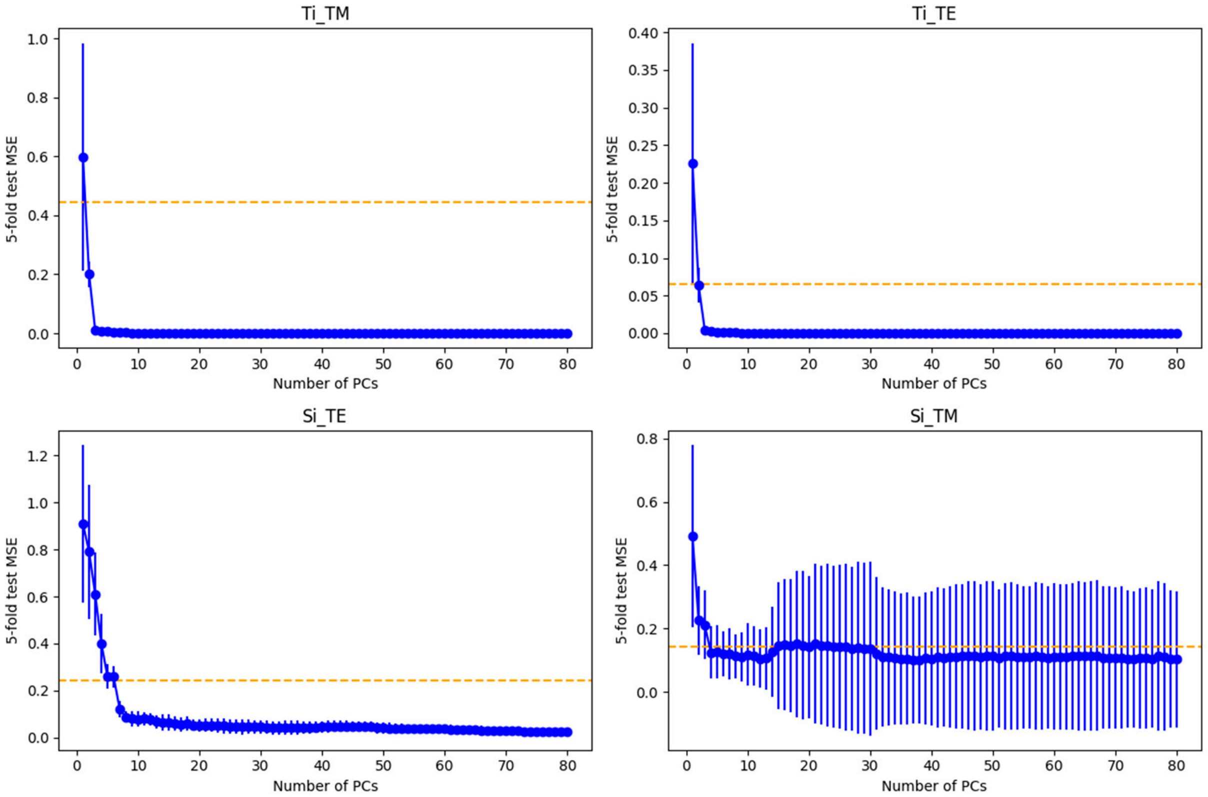


Supplementary Figure S1: Contribution of additional principal components to the hold-out MSE across 5-fold cross validation across the Ti-TM (top-left), Ti-TE (top-right), Si-TE (bottom-left), and Si-TM (bottom-right). The x-axis represents the integer number of principal components added to the model and the y-axis represents the mean MSE for the hold-out set. Vertical bars represent the standard deviation of the hold-out MSE across the 5 folds. The horizontal orange line represents the mean hold-out MSE when only the best single predictor was used.

The PCA model was fit on the training data only (which is why it is capped at only 80 features). The hold-out data was transformed into the PC space generated by the training data. Since PC space is generated without knowledge of the labels. The first principal component may not capture the most relevant dimension to the labels. The order of the PCs reflects the variation explained from the training data. But for the first 3 datasets we can see that the first few PCs capture most of the signal associated with labels and generally outperform the best single predictor with only marginal improvements in MSE when more PCs are added. We have also included supplemental Table 3, which includes the full results from the figure, and we were able to observe that the optimal number of principal components for the Ti-TM dataset is 35, 77 principal components for the Ti-TE dataset, 80 principal components for the Si-TE dataset, and 37 principal components for the Si-TM dataset.

# Non-linear modeling results

To study the effects of non-linear machine learning models across the datasets, we decided to evaluate the performance of the following models: Support Vector Regression (SVR) with radial basis, multi-layer perceptron (neural network), and eXtreme Gradient Boosting (XGBoost) regressor. Models were first fit using their default hyperparameters on the 80 principal components generated on the dataset during the 5-fold cross-validation. MSE values for the hold-out set were averaged, and the results can be found in Supplementary Table 4. Using the default parameters we observed increased MSE values for all models across Si and Ti datasets regardless of polarization. We then tried to tune the hyperparameters for the 3 non-linear machine learning models using 100 iterations of Tree Parzen Estimation (TPE) hyperparameter sampling implemented through optuna. Optimal hyperparameters as determined by optuna are also described in Supplementary Table 4. We observed that hyperparameter tuning improved model performance; however, for most datasets the performance improvement did not achieve lower mean MSE on the cross-validated hold-out sets. With the exception of optimized SVR with radial basis kernel on the Si-TM dataset which achieved a ~2.28-fold reduction in MSE compared to the linear modeling approach.
